# Supplementary figures and images for: Ubiquitination and degradation of NF90 by Tim-3 inhibits antiviral innate immunity (part 1 of 2)
Source: eLife. 2021 Jun 10;10:e66501. doi: 10.7554/eLife.66501 (PMC8225388; doi:10.7554/eLife.66501)

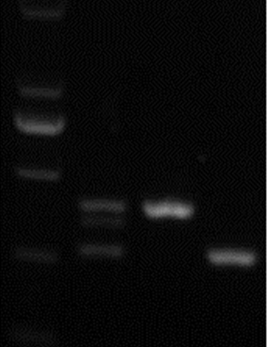

Supplement: Figure 1—source code 1. [file elife-66501-fig1-code1.zip › Figure 1í¬source code 1-Related to Figure 1D&E/B.png]

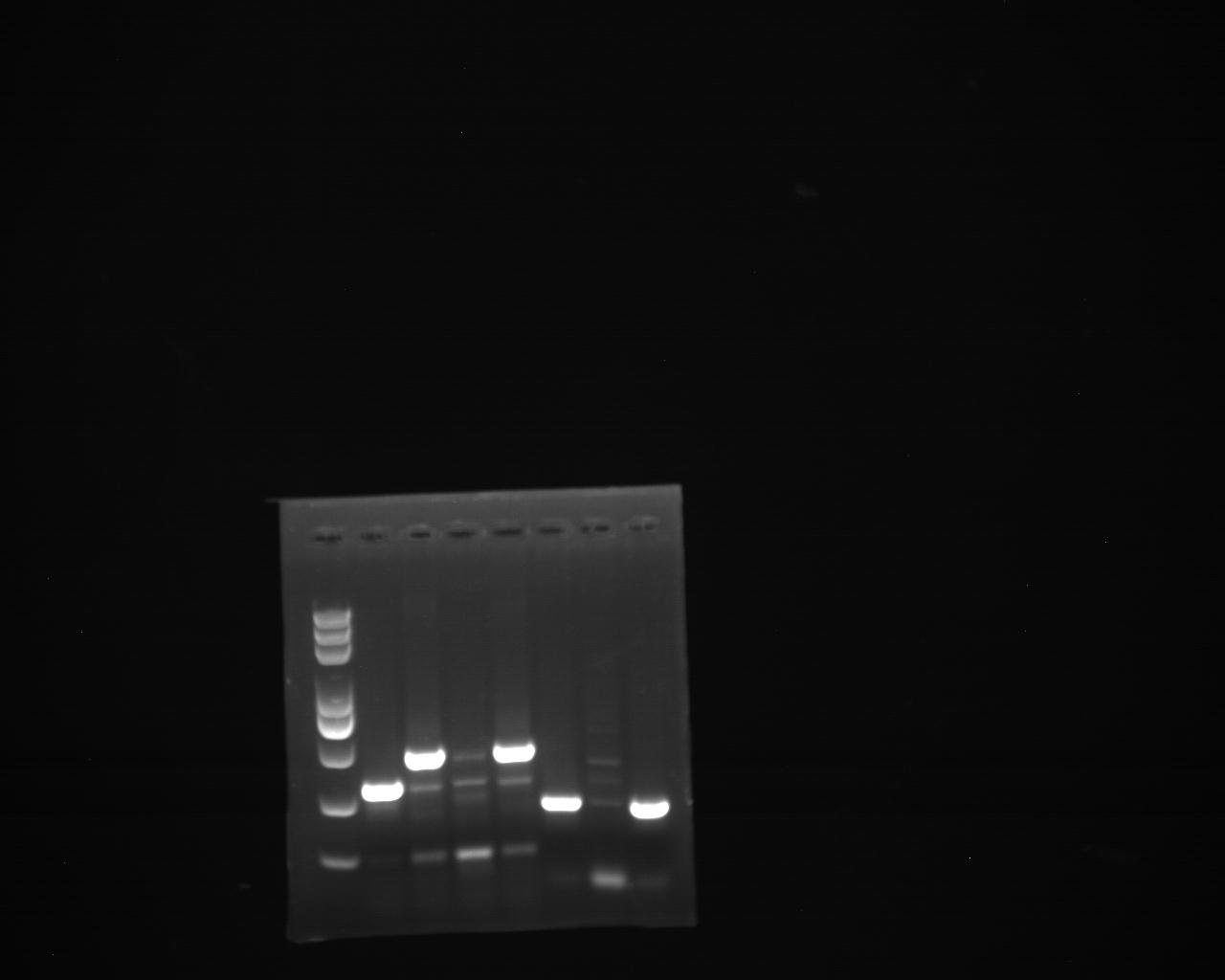

Supplement: Figure 1—source code 2. [file elife-66501-fig1-code2.zip › Figure 1í¬source code 2-Related to Figure 1D&E/C.jpg]

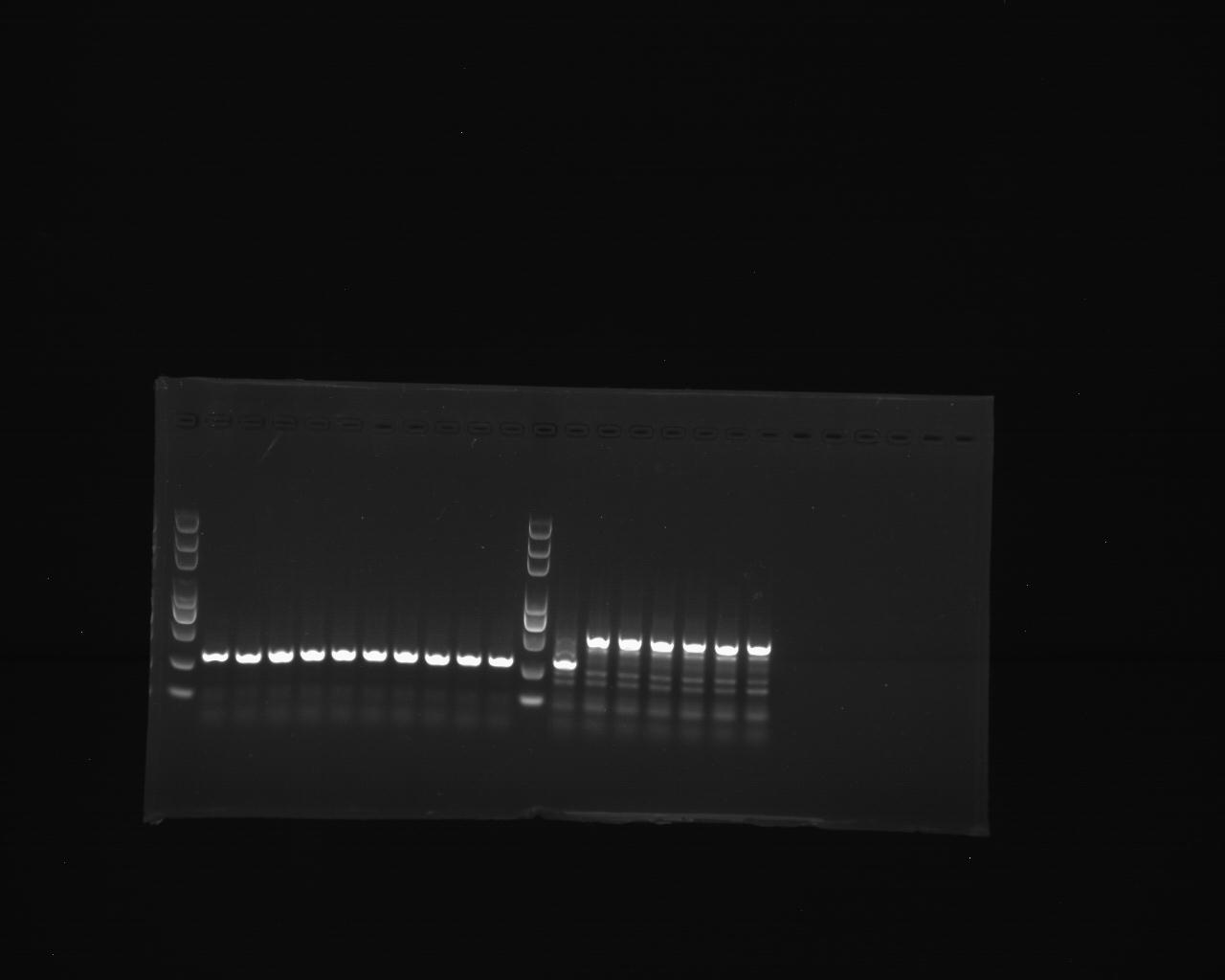

Supplement: Figure 1—source code 3. [file elife-66501-fig1-code3.zip › Figure 1í¬source code 3-Related to Figure 1D&E/D.jpg]

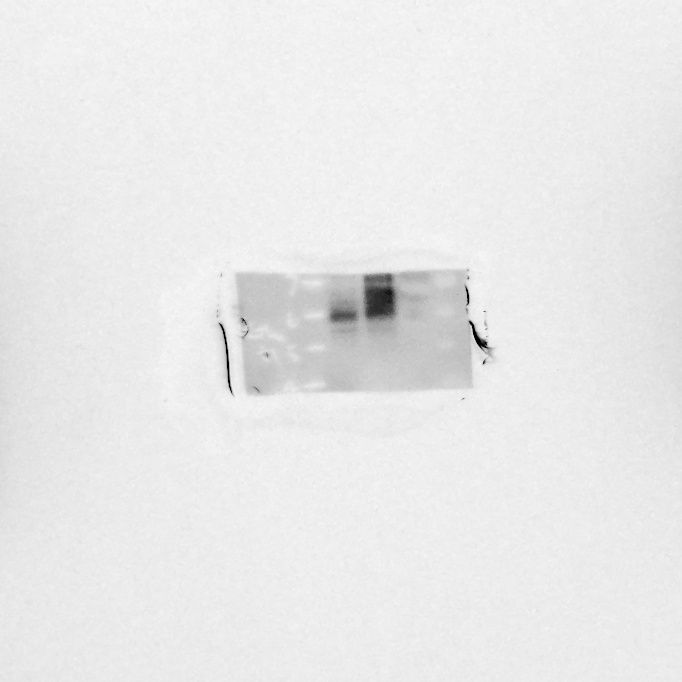

Supplement: Figure 1—source code 4. [file elife-66501-fig1-code4.zip › Figure 1í¬source code 4-Related to Figure 1D&E/E-Tim-3.jpg]

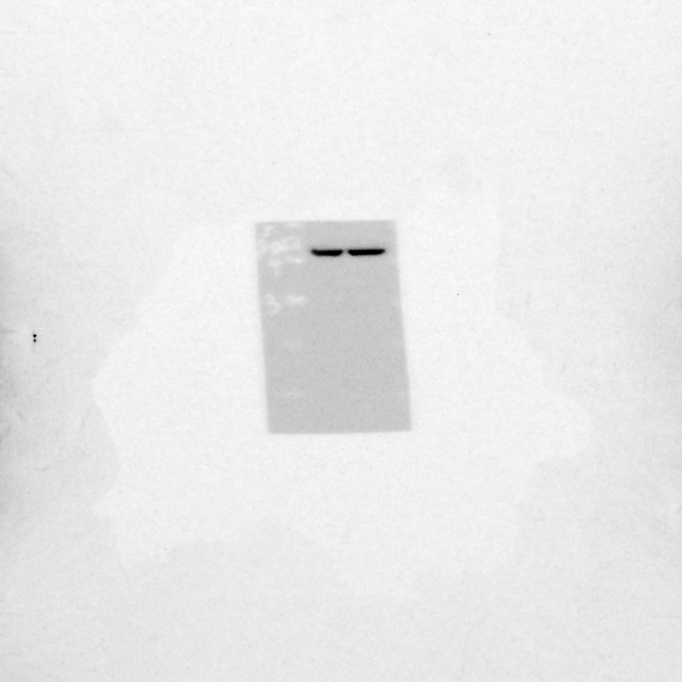

Supplement: Figure 1—source code 4. [file elife-66501-fig1-code4.zip › Figure 1í¬source code 4-Related to Figure 1D&E/E-a┬-actin.jpg]

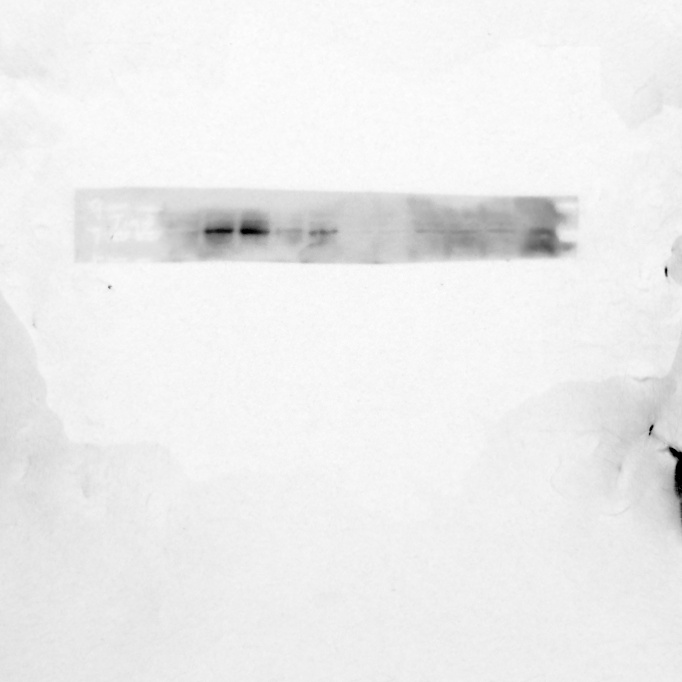

Supplement: Figure 1—source data 2. [file elife-66501-fig1-data2.zip › Figure1-source data2-Related to Figure1B/Fig1B-Tim-3.jpg]

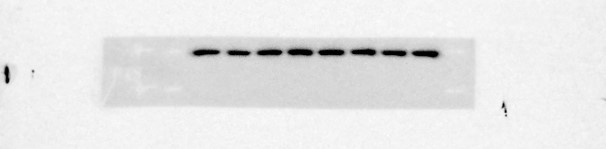

Supplement: Figure 1—source data 2. [file elife-66501-fig1-data2.zip › Figure1-source data2-Related to Figure1B/Fig1B-a┬-actin.jpg]

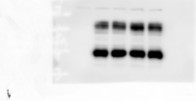

Supplement: Figure 1—source data 3. [file elife-66501-fig1-data3.zip › Figure1-source data3-Related to Figure1C/Fig1C-Input-IB-HA.jpg]

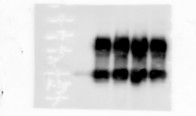

Supplement: Figure 1—source data 3. [file elife-66501-fig1-data3.zip › Figure1-source data3-Related to Figure1C/Fig1C-IP-IB-HA.jpg]

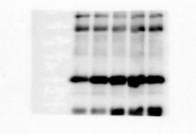

Supplement: Figure 1—source data 3. [file elife-66501-fig1-data3.zip › Figure1-source data3-Related to Figure1C/Fig1C-IP-IB-p-Tyr.jpg]

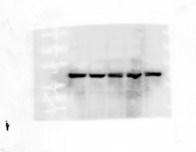

Supplement: Figure 1—source data 3. [file elife-66501-fig1-data3.zip › Figure1-source data3-Related to Figure1C/Fig1C-a┬-actin.jpg]

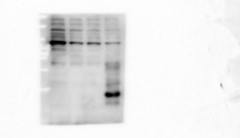

Supplement: Figure 2—source code 1. [file elife-66501-fig2-code1.zip › Figure 2í¬source code 1-Related to Figure 2A&B/A-Tim-3.jpg]

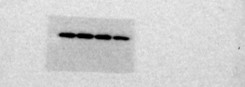

Supplement: Figure 2—source code 1. [file elife-66501-fig2-code1.zip › Figure 2í¬source code 1-Related to Figure 2A&B/A-a┬-actin.jpg]

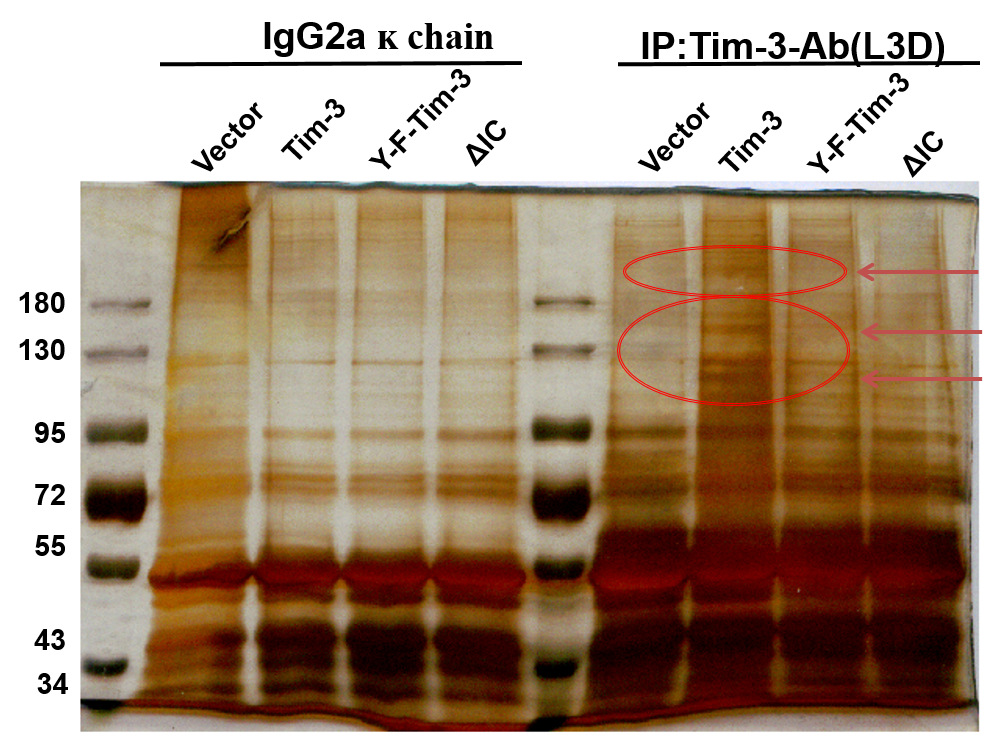

Supplement: Figure 2—source code 2. [file elife-66501-fig2-code2.zip › Figure 2í¬source code 2-Related to Figure 2A&B/B.png]

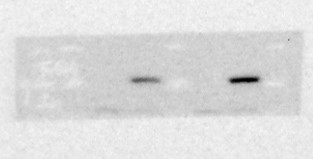

Supplement: Figure 2—source data 1. [file elife-66501-fig2-data1.zip › Figure2-source data1-Related to Figure2A/Fig2A-Input-IB-Flag.jpg]

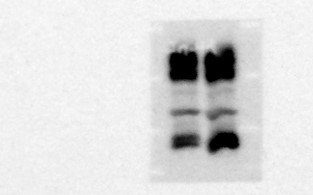

Supplement: Figure 2—source data 1. [file elife-66501-fig2-data1.zip › Figure2-source data1-Related to Figure2A/Fig2A-Input-IB-HA.jpg]

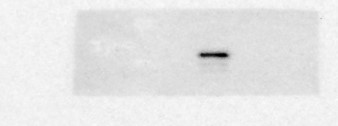

Supplement: Figure 2—source data 1. [file elife-66501-fig2-data1.zip › Figure2-source data1-Related to Figure2A/Fig2A-IP-IB-Flag.jpg]

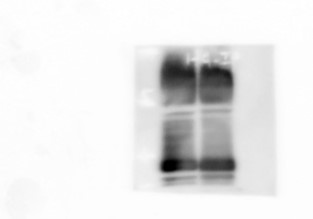

Supplement: Figure 2—source data 1. [file elife-66501-fig2-data1.zip › Figure2-source data1-Related to Figure2A/Fig2A-IP-IB-HA.jpg]

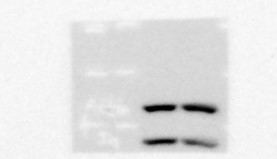

Supplement: Figure 2—source data 1. [file elife-66501-fig2-data1.zip › Figure2-source data1-Related to Figure2A/Fig2A-a┬-actin.jpg]

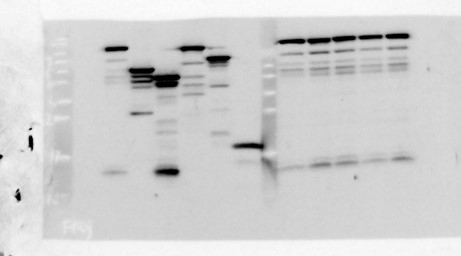

Supplement: Figure 2—source data 2. [file elife-66501-fig2-data2.zip › Figure2-source data2-Related to Figure2B/Fig2B-Input-IB-Flag.jpg]

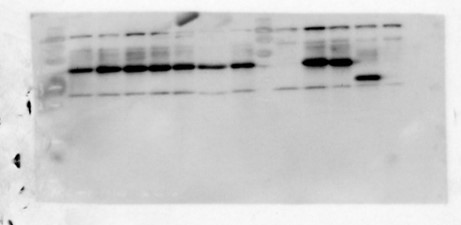

Supplement: Figure 2—source data 2. [file elife-66501-fig2-data2.zip › Figure2-source data2-Related to Figure2B/Fig2B-Input-IB-Tim-3.jpg]

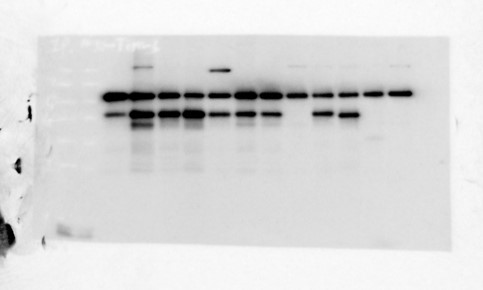

Supplement: Figure 2—source data 2. [file elife-66501-fig2-data2.zip › Figure2-source data2-Related to Figure2B/Fig2B-IP-IB-Flag.jpg]

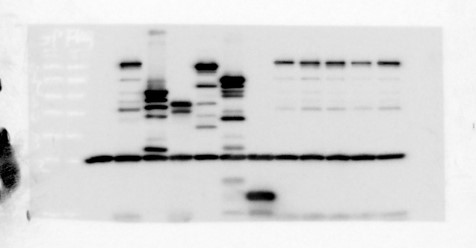

Supplement: Figure 2—source data 2. [file elife-66501-fig2-data2.zip › Figure2-source data2-Related to Figure2B/Fig2B-IP-IB-Tim-3.jpg]

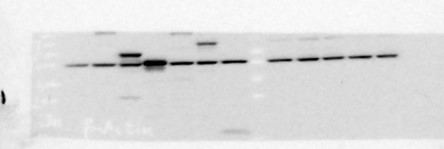

Supplement: Figure 2—source data 2. [file elife-66501-fig2-data2.zip › Figure2-source data2-Related to Figure2B/Fig2B-a┬-actin.jpg]

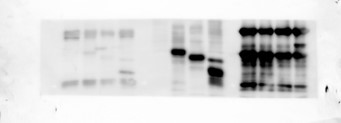

Supplement: Figure 2—source data 3. [file elife-66501-fig2-data3.zip › Figure2-source data3-Related to Figure2D/Fig2D-Input-IB-Tim-3.jpg]

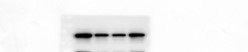

Supplement: Figure 2—source data 3. [file elife-66501-fig2-data3.zip › Figure2-source data3-Related to Figure2D/Fig2D-Input-IB-V5.jpg]

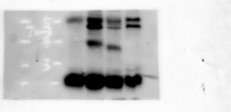

Supplement: Figure 2—source data 3. [file elife-66501-fig2-data3.zip › Figure2-source data3-Related to Figure2D/Fig2D-IP-IB-Tim-3.jpg]

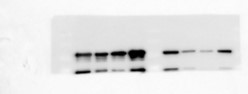

Supplement: Figure 2—source data 3. [file elife-66501-fig2-data3.zip › Figure2-source data3-Related to Figure2D/Fig2D-IP-IB-V5.jpg]

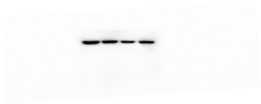

Supplement: Figure 2—source data 3. [file elife-66501-fig2-data3.zip › Figure2-source data3-Related to Figure2D/Fig2D-a┬-actin.jpg]

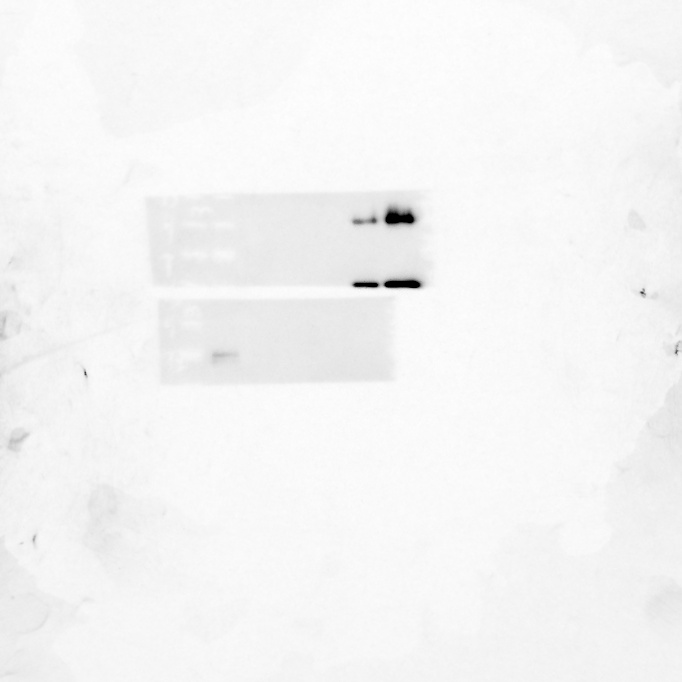

Supplement: Figure 2—source data 4. [file elife-66501-fig2-data4.zip › Figure2-source data4-Related to Figure2E/Fig2E-NF90.jpg]

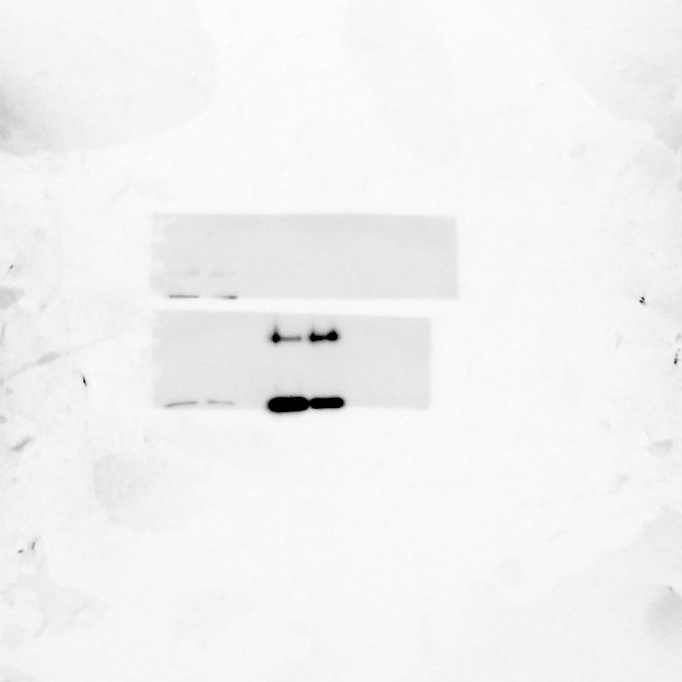

Supplement: Figure 2—source data 4. [file elife-66501-fig2-data4.zip › Figure2-source data4-Related to Figure2E/Fig2E-WT-NF90.jpg]

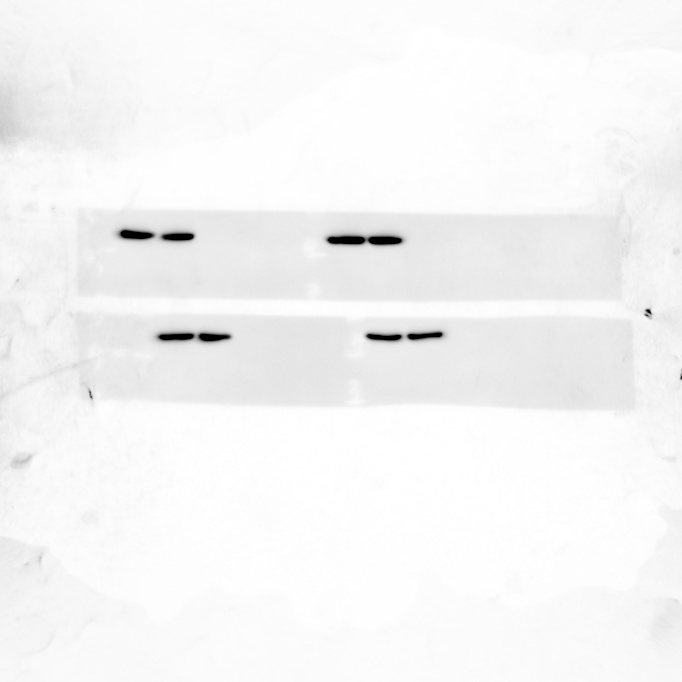

Supplement: Figure 2—source data 4. [file elife-66501-fig2-data4.zip › Figure2-source data4-Related to Figure2E/Fig2E-a┬-actin.jpg]

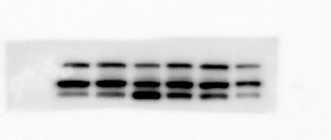

Supplement: Figure 3—source code 1. [file elife-66501-fig3-code1.zip › Figure 3í¬source code 1-Related to Figure 3A/Input-IB-NF90.jpg]

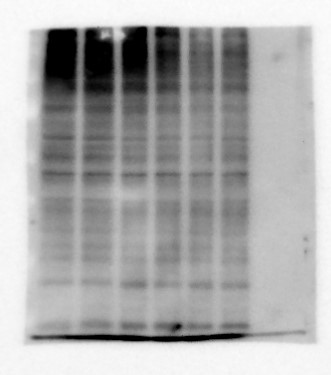

Supplement: Figure 3—source code 1. [file elife-66501-fig3-code1.zip › Figure 3í¬source code 1-Related to Figure 3A/Input-IB-Ub.jpg]

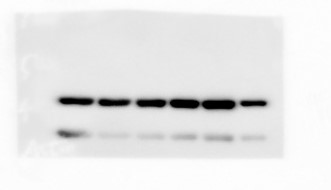

Supplement: Figure 3—source code 1. [file elife-66501-fig3-code1.zip › Figure 3í¬source code 1-Related to Figure 3A/Input-a┬-actin.jpg]

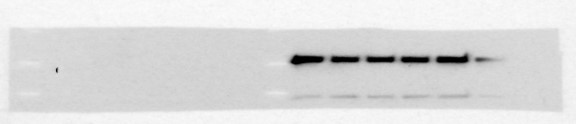

Supplement: Figure 3—source data 1. [file elife-66501-fig3-data1.zip › Figure3-source data1-Related to Figure3A/Fig3A-Input-IB-NF-90.jpg]

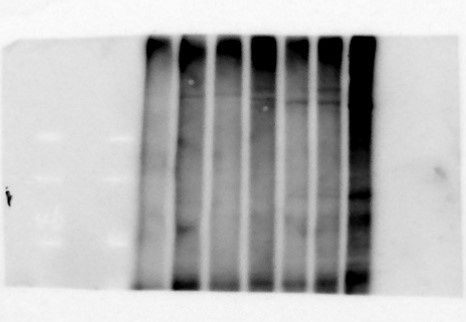

Supplement: Figure 3—source data 1. [file elife-66501-fig3-data1.zip › Figure3-source data1-Related to Figure3A/Fig3A-Input-IB-Ub.jpg]

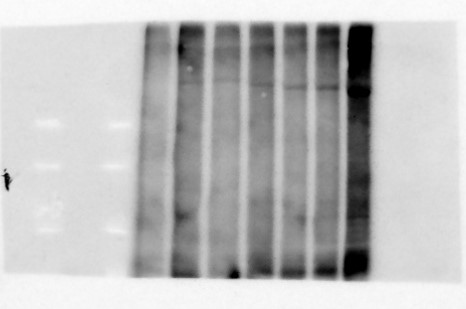

Supplement: Figure 3—source data 1. [file elife-66501-fig3-data1.zip › Figure3-source data1-Related to Figure3A/Fig3A-Input-IB-Ub-k48.jpg]

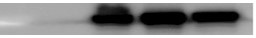

Supplement: Figure 3—source data 2. [file elife-66501-fig3-data2.zip › Figure3-source data2-Related to Figure3B/Fig3B-Input-IB-Flag.jpg]

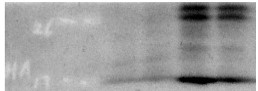

Supplement: Figure 3—source data 2. [file elife-66501-fig3-data2.zip › Figure3-source data2-Related to Figure3B/Fig3B-Input-IB-HA.jpg]

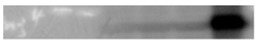

Supplement: Figure 3—source data 2. [file elife-66501-fig3-data2.zip › Figure3-source data2-Related to Figure3B/Fig3B-Input-IB-Tim-3.jpg]

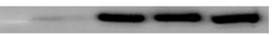

Supplement: Figure 3—source data 2. [file elife-66501-fig3-data2.zip › Figure3-source data2-Related to Figure3B/Fig3B-IP-IB-Flag.jpg]

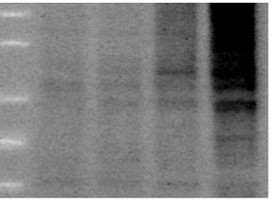

Supplement: Figure 3—source data 2. [file elife-66501-fig3-data2.zip › Figure3-source data2-Related to Figure3B/Fig3B-IP-IB-HA.jpg]

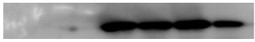

Supplement: Figure 3—source data 2. [file elife-66501-fig3-data2.zip › Figure3-source data2-Related to Figure3B/Fig3B-a┬-actin.jpg]

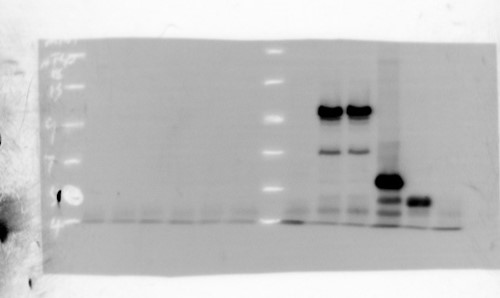

Supplement: Figure 3—source data 3. [file elife-66501-fig3-data3.zip › Figure3-source data3-Related to Figure3D/Fig3D-Input-IB-Flag.jpg]

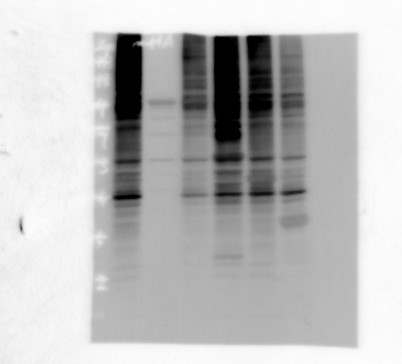

Supplement: Figure 3—source data 3. [file elife-66501-fig3-data3.zip › Figure3-source data3-Related to Figure3D/Fig3D-Input-IB-HA.jpg]

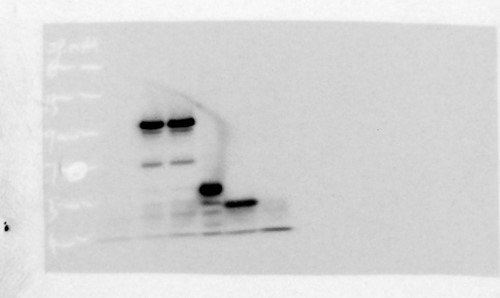

Supplement: Figure 3—source data 3. [file elife-66501-fig3-data3.zip › Figure3-source data3-Related to Figure3D/Fig3D-IP-IB-Flag.jpg]

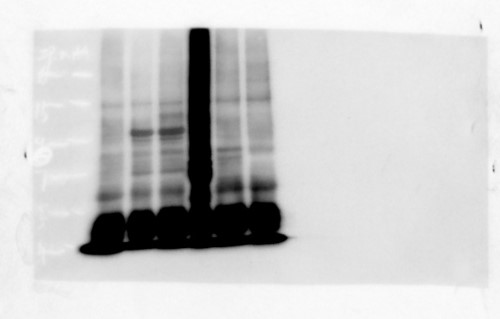

Supplement: Figure 3—source data 3. [file elife-66501-fig3-data3.zip › Figure3-source data3-Related to Figure3D/Fig3D-IP-IB-HA.jpg]

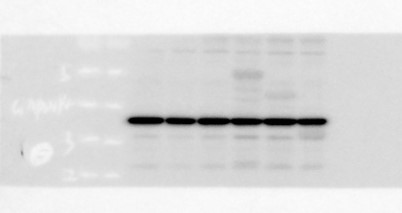

Supplement: Figure 3—source data 3. [file elife-66501-fig3-data3.zip › Figure3-source data3-Related to Figure3D/Fig3D-a┬-actin.jpg]

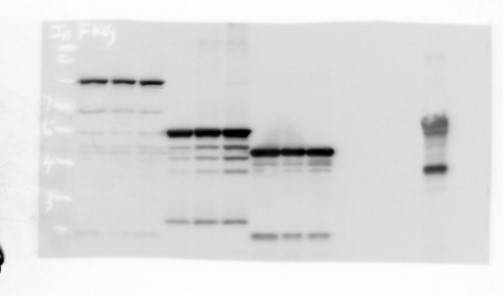

Supplement: Figure 3—source data 4. [file elife-66501-fig3-data4.zip › Figure3-source data4-Related to Figure3E/Fig3E-Input-IB-Flag.jpg]

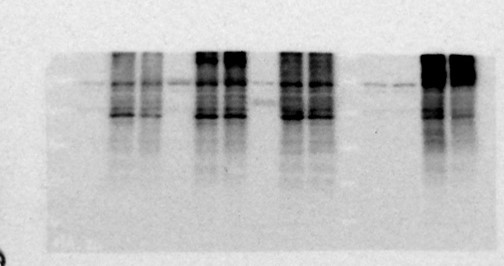

Supplement: Figure 3—source data 4. [file elife-66501-fig3-data4.zip › Figure3-source data4-Related to Figure3E/Fig3E-Input-IB-HA.jpg]

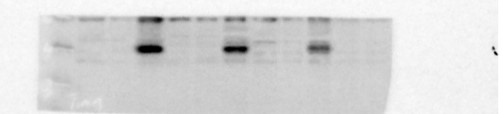

Supplement: Figure 3—source data 4. [file elife-66501-fig3-data4.zip › Figure3-source data4-Related to Figure3E/Fig3E-Input-IB-Tim-3.jpg]

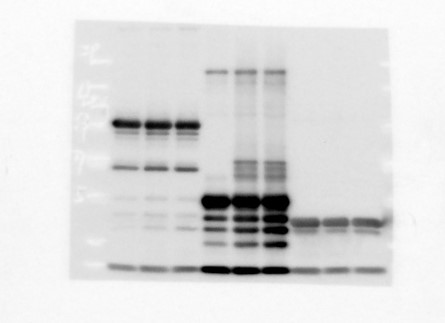

Supplement: Figure 3—source data 4. [file elife-66501-fig3-data4.zip › Figure3-source data4-Related to Figure3E/Fig3E-IP-IB-Flag.jpg]

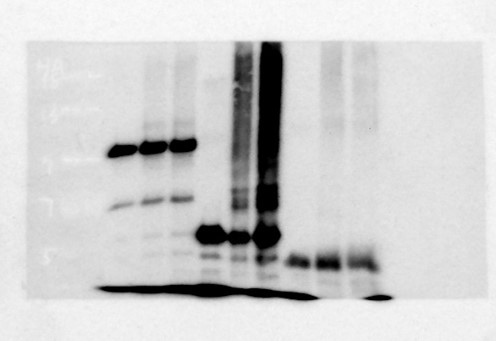

Supplement: Figure 3—source data 4. [file elife-66501-fig3-data4.zip › Figure3-source data4-Related to Figure3E/Fig3E-IP-IB-HA.jpg]

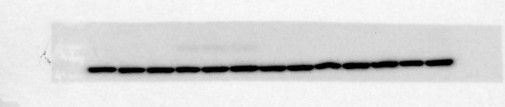

Supplement: Figure 3—source data 4. [file elife-66501-fig3-data4.zip › Figure3-source data4-Related to Figure3E/Fig3E-a┬-actin.jpg]

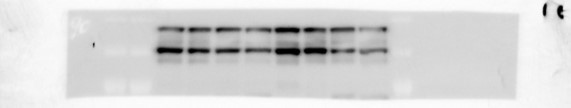

Supplement: Figure 4—source code 2. [file elife-66501-fig4-code2.zip › Figure 4í¬source code 2-Related to Figure 4B/B-NF90.jpg]

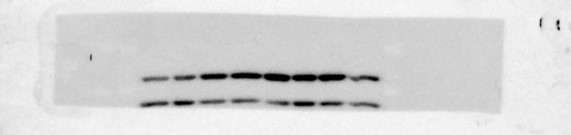

Supplement: Figure 4—source code 2. [file elife-66501-fig4-code2.zip › Figure 4í¬source code 2-Related to Figure 4B/B-a┬-actin.jpg]

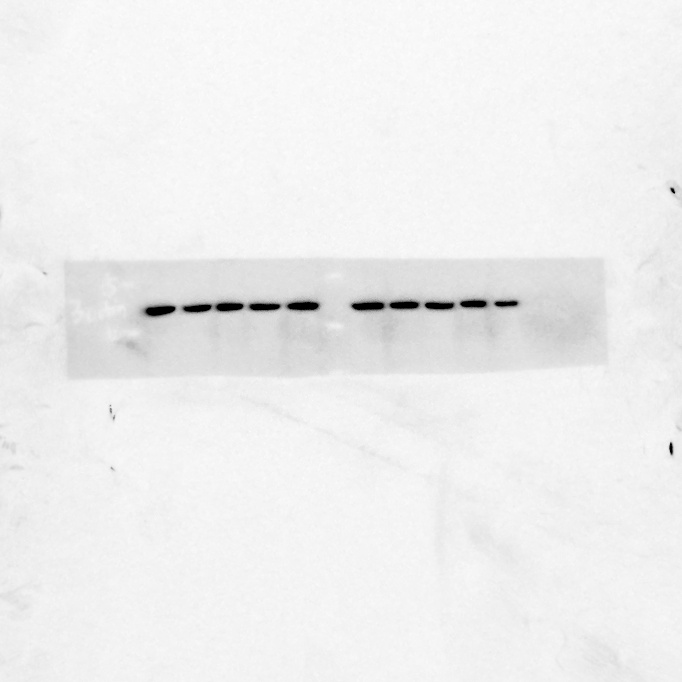

Supplement: Figure 4—source data 1. [file elife-66501-fig4-data1.zip › Figure4-source data1-Related to Figure4B/Fig 4B -a┬-actin.jpg]

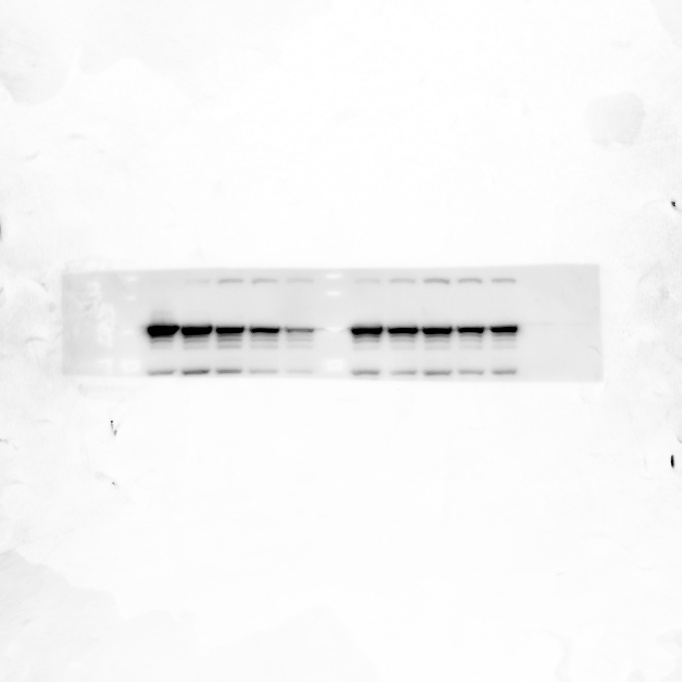

Supplement: Figure 4—source data 1. [file elife-66501-fig4-data1.zip › Figure4-source data1-Related to Figure4B/Fig 4B-Flag-MG132+.jpg]

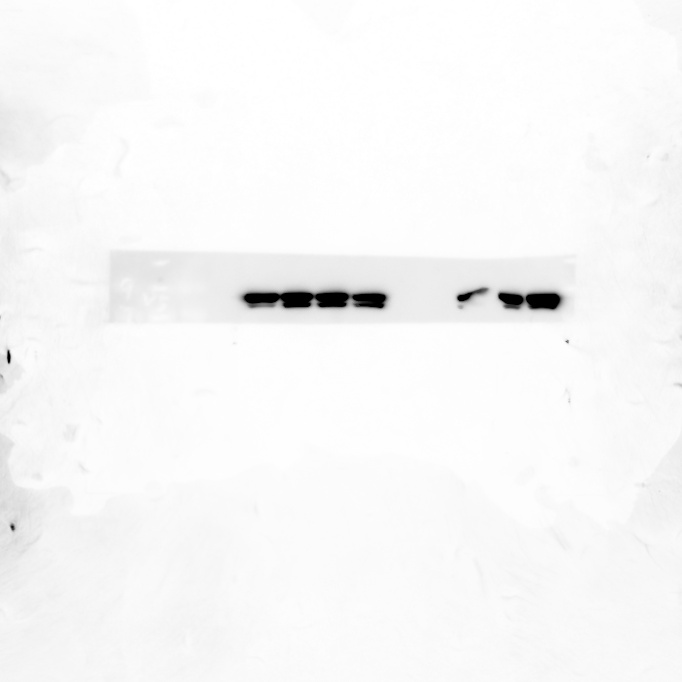

Supplement: Figure 4—source data 1. [file elife-66501-fig4-data1.zip › Figure4-source data1-Related to Figure4B/Fig 4B-V5.jpg]

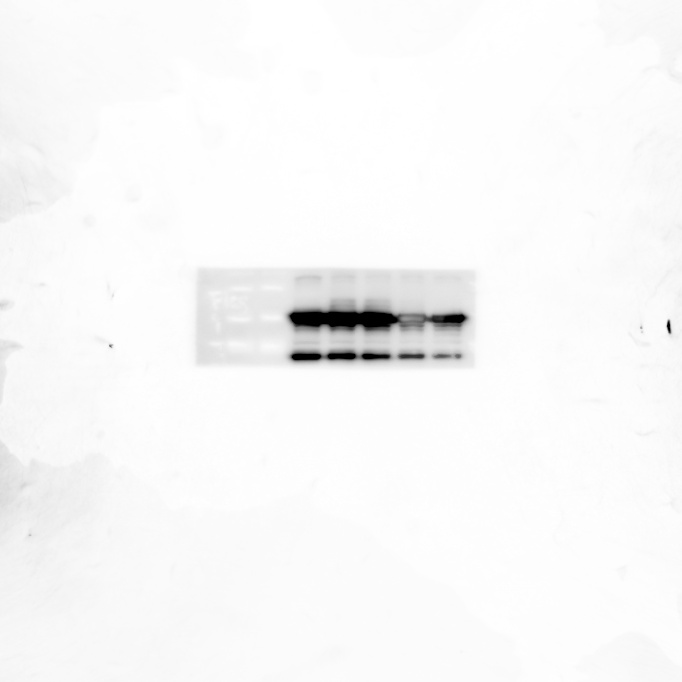

Supplement: Figure 4—source data 1. [file elife-66501-fig4-data1.zip › Figure4-source data1-Related to Figure4B/Fig4 B-NF90-Flag MG132-.jpg]

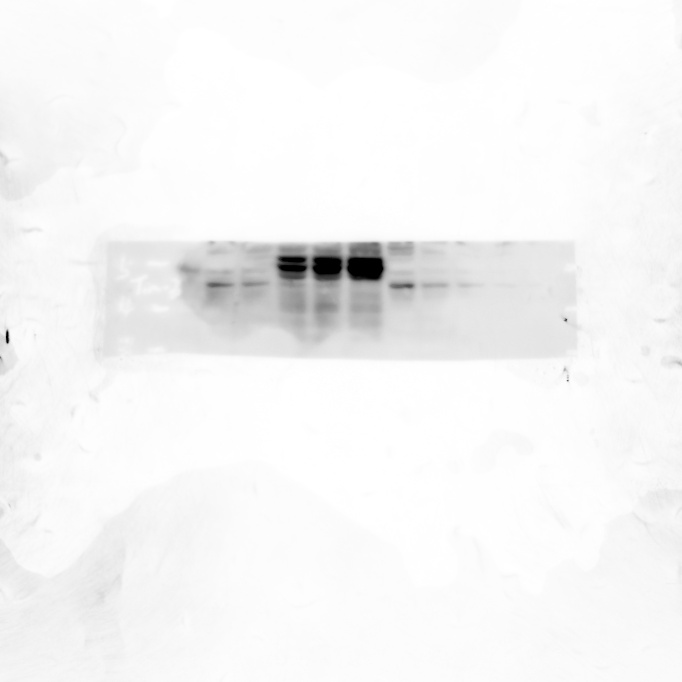

Supplement: Figure 4—source data 2. [file elife-66501-fig4-data2.zip › Figure4-source data2-Related to Figure4C/Fig 4C-Tim-3.jpg]

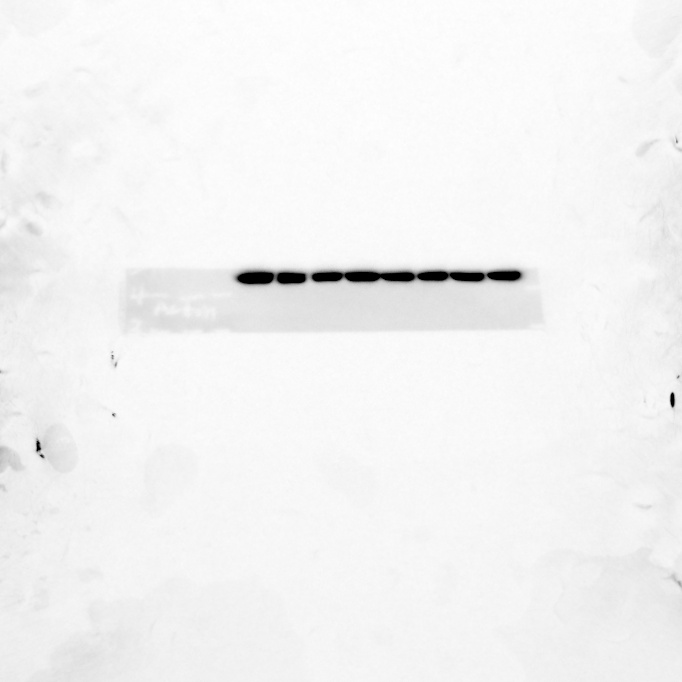

Supplement: Figure 4—source data 3. [file elife-66501-fig4-data3.zip › Figure4-source data3-Related to Figure4D/Fig4 D-a┬-actin.jpg]

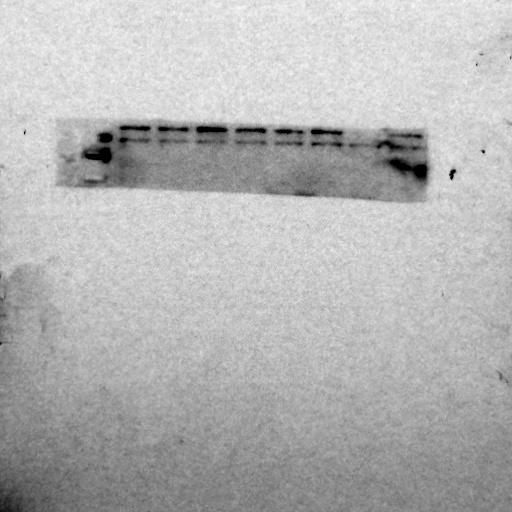

Supplement: Figure 4—source data 3. [file elife-66501-fig4-data3.zip › Figure4-source data3-Related to Figure4D/Fig4D-TRIM47.jpg]

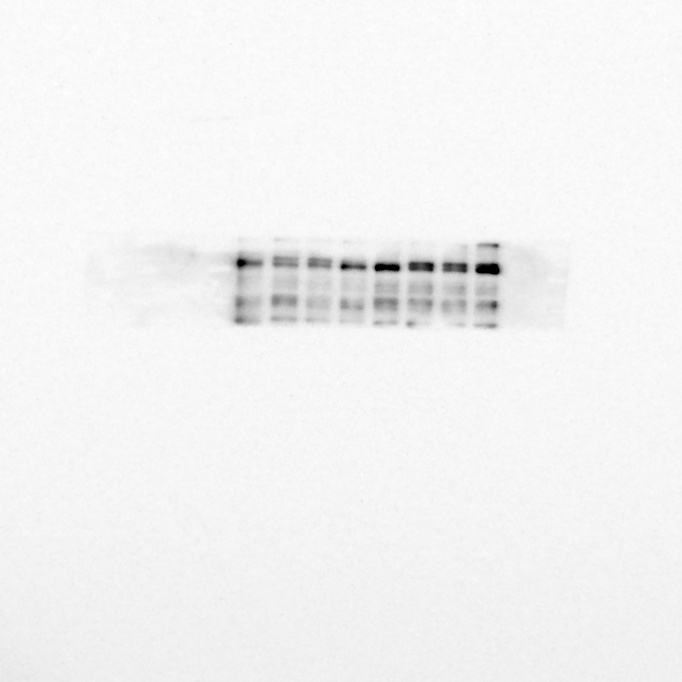

Supplement: Figure 4—source data 4. [file elife-66501-fig4-data4.zip › Figure4-source data4-Related to Figure4E/Fig4E-TRIM47.jpg]

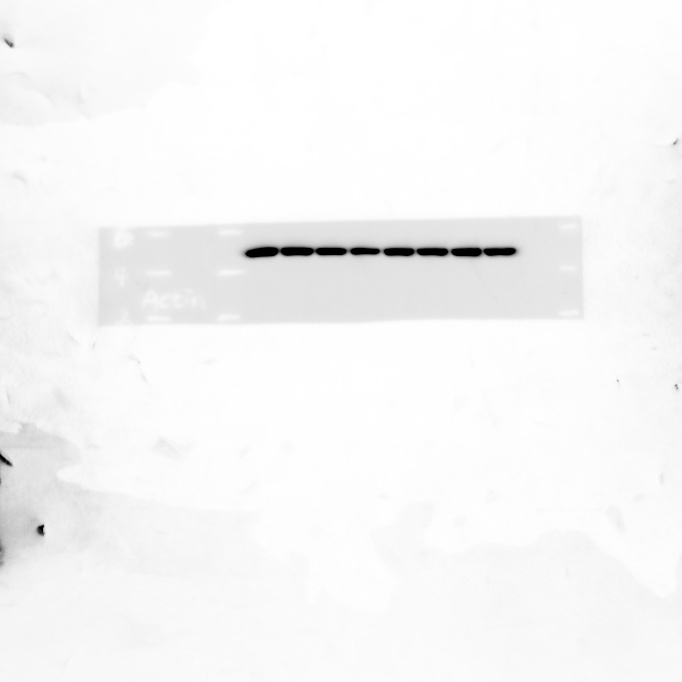

Supplement: Figure 4—source data 4. [file elife-66501-fig4-data4.zip › Figure4-source data4-Related to Figure4E/Fig4E-a┬-actin.jpg]

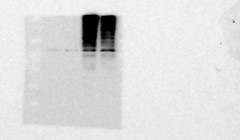

Supplement: Figure 4—source data 5. [file elife-66501-fig4-data5.zip › Figure4-source data5-Related to Figure4F/Fig4F-Input-IB-HA.jpg]

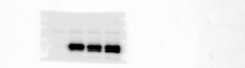

Supplement: Figure 4—source data 5. [file elife-66501-fig4-data5.zip › Figure4-source data5-Related to Figure4F/Fig4F-Input-IB-V5.jpg]

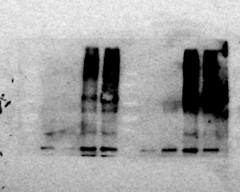

Supplement: Figure 4—source data 5. [file elife-66501-fig4-data5.zip › Figure4-source data5-Related to Figure4F/Fig4F-IP-IB-HA.jpg]

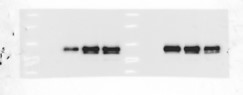

Supplement: Figure 4—source data 5. [file elife-66501-fig4-data5.zip › Figure4-source data5-Related to Figure4F/Fig4F-IP-IB-V5.jpg]

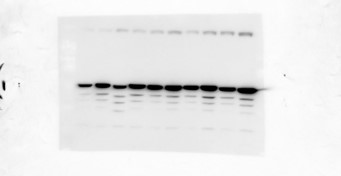

Supplement: Figure 5—source code 1. [file elife-66501-fig5-code1.zip › Figure 5í¬source code 1-Related to Figure 5E/Input-IB-Flag.jpg]

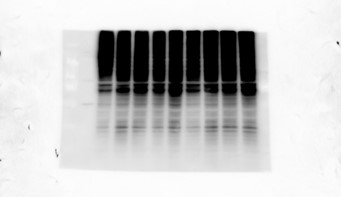

Supplement: Figure 5—source code 1. [file elife-66501-fig5-code1.zip › Figure 5í¬source code 1-Related to Figure 5E/Input-IB-HA.jpg]

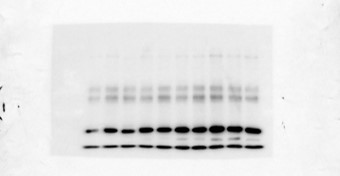

Supplement: Figure 5—source code 1. [file elife-66501-fig5-code1.zip › Figure 5í¬source code 1-Related to Figure 5E/IP-IB-Flag.jpg]

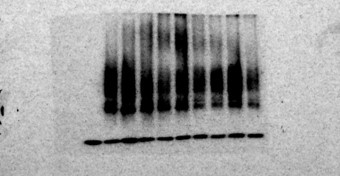

Supplement: Figure 5—source code 1. [file elife-66501-fig5-code1.zip › Figure 5í¬source code 1-Related to Figure 5E/IP-IB-HA.jpg]

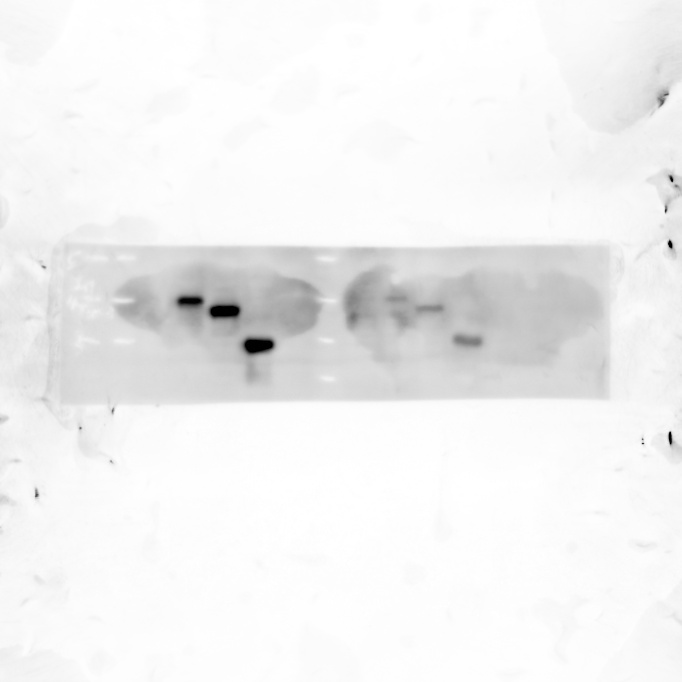

Supplement: Figure 5—source data 1. [file elife-66501-fig5-data1.zip › Figure5-source data1-Related to Figure5A/Fig 5A-Input-IB-Tim-3.jpg]

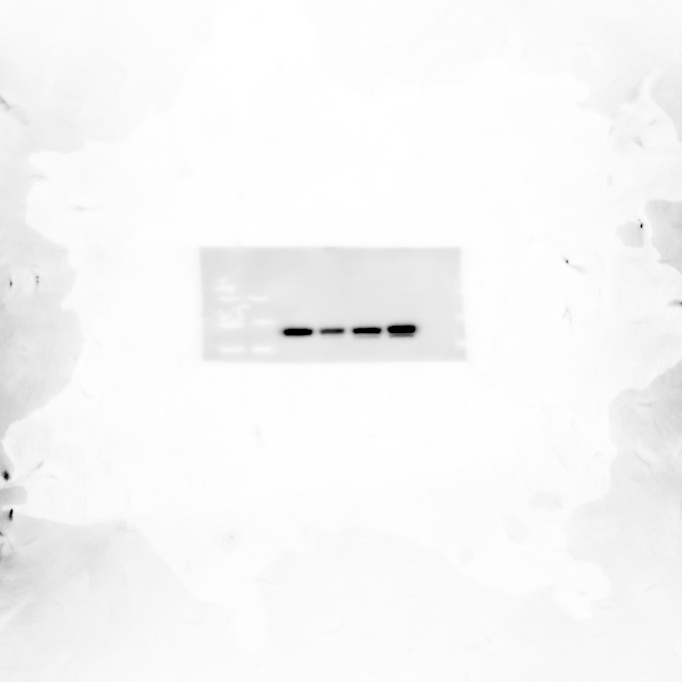

Supplement: Figure 5—source data 1. [file elife-66501-fig5-data1.zip › Figure5-source data1-Related to Figure5A/Fig 5A-Input-IB-V5.jpg]

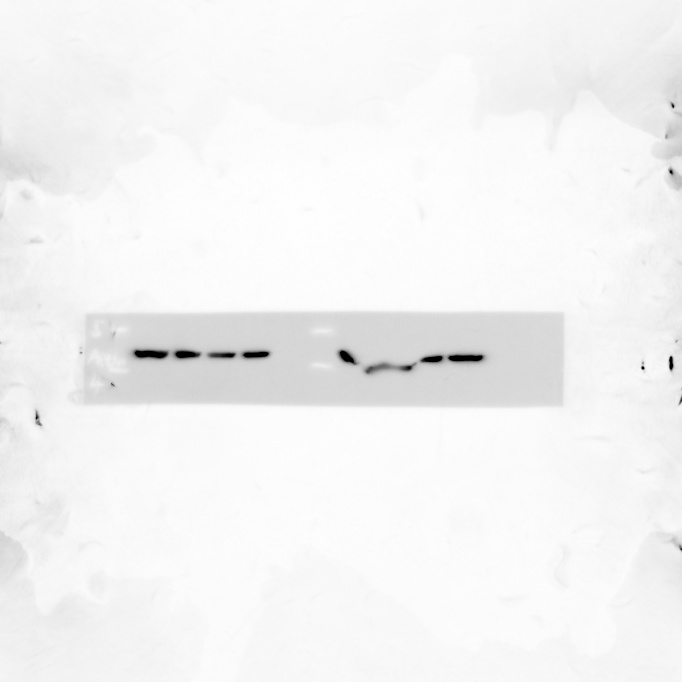

Supplement: Figure 5—source data 1. [file elife-66501-fig5-data1.zip › Figure5-source data1-Related to Figure5A/Fig 5A-a┬-actin.jpg]

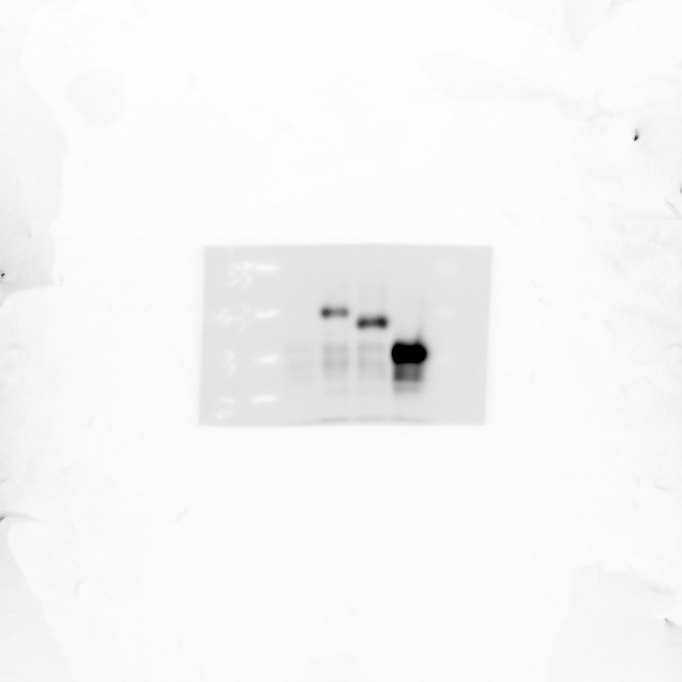

Supplement: Figure 5—source data 1. [file elife-66501-fig5-data1.zip › Figure5-source data1-Related to Figure5A/Fig5A-IP-IB-Tim-3.jpg]

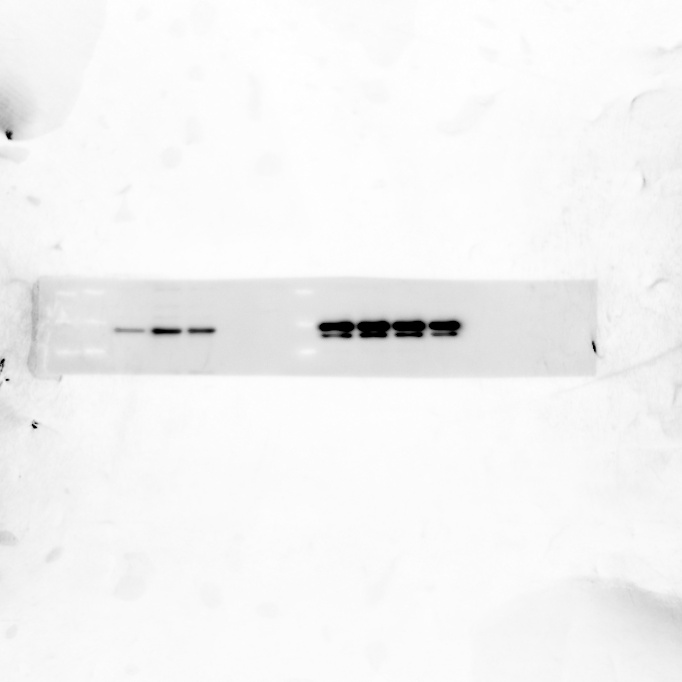

Supplement: Figure 5—source data 1. [file elife-66501-fig5-data1.zip › Figure5-source data1-Related to Figure5A/Fig5A-IP-IB-V5.jpg]

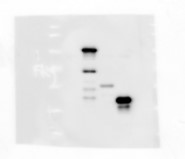

Supplement: Figure 5—source data 2. [file elife-66501-fig5-data2.zip › Figure5-source data2-Related to Figure5B/Fig5B-Input-IB-Flag.jpg]

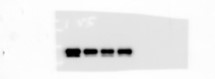

Supplement: Figure 5—source data 2. [file elife-66501-fig5-data2.zip › Figure5-source data2-Related to Figure5B/Fig5B-Input-IB-V5.jpg]

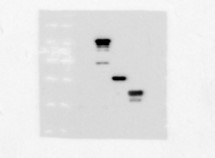

Supplement: Figure 5—source data 2. [file elife-66501-fig5-data2.zip › Figure5-source data2-Related to Figure5B/Fig5B-IP-IB-Flag.jpg]

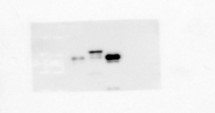

Supplement: Figure 5—source data 2. [file elife-66501-fig5-data2.zip › Figure5-source data2-Related to Figure5B/Fig5B-IP-IB-V5.jpg]

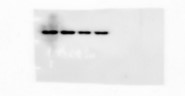

Supplement: Figure 5—source data 2. [file elife-66501-fig5-data2.zip › Figure5-source data2-Related to Figure5B/Fig5B-a┬-actin.jpg]

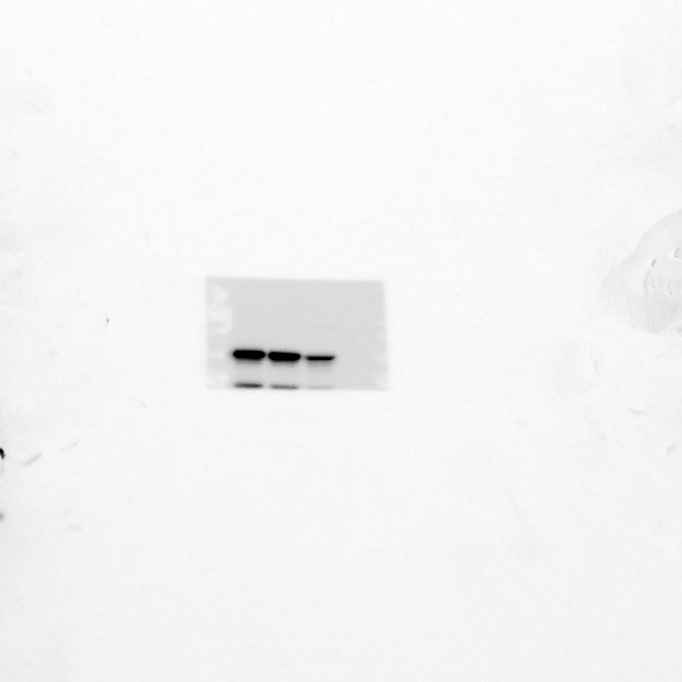

Supplement: Figure 5—source data 3. [file elife-66501-fig5-data3.zip › Figure5-source data3-Related to Figure5C/Fig5C Input -IB-Flag.jpg]

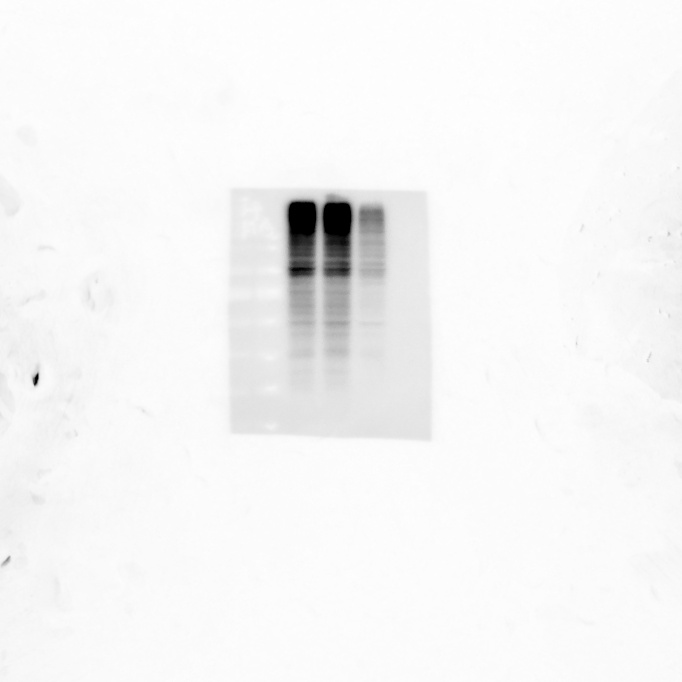

Supplement: Figure 5—source data 3. [file elife-66501-fig5-data3.zip › Figure5-source data3-Related to Figure5C/Fig5C Input-IB HA-Ub-K48.jpg]

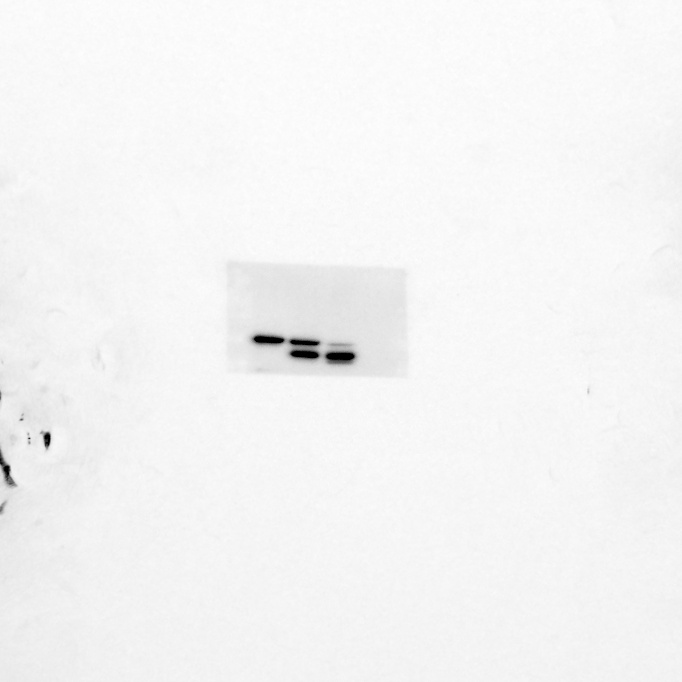

Supplement: Figure 5—source data 3. [file elife-66501-fig5-data3.zip › Figure5-source data3-Related to Figure5C/Fig5C Input-IB- V5.jpg]

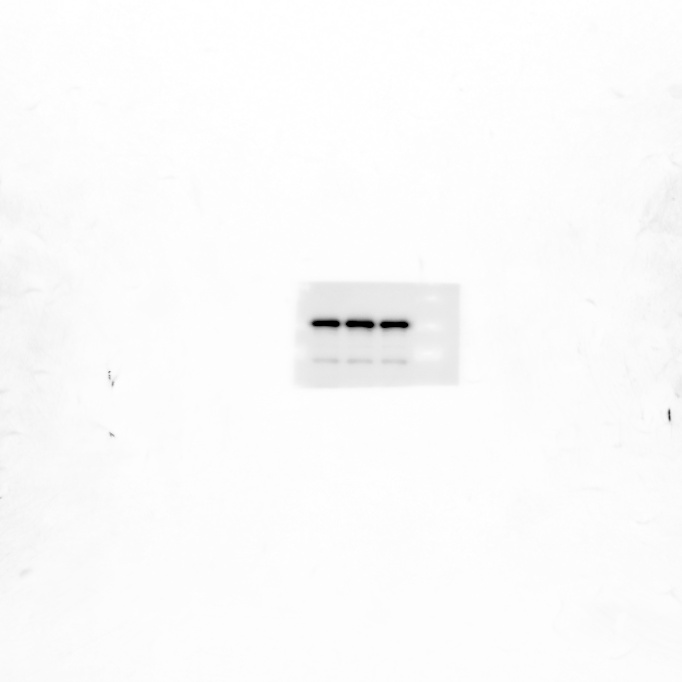

Supplement: Figure 5—source data 3. [file elife-66501-fig5-data3.zip › Figure5-source data3-Related to Figure5C/Fig5C IP-IB-Flag.jpg]

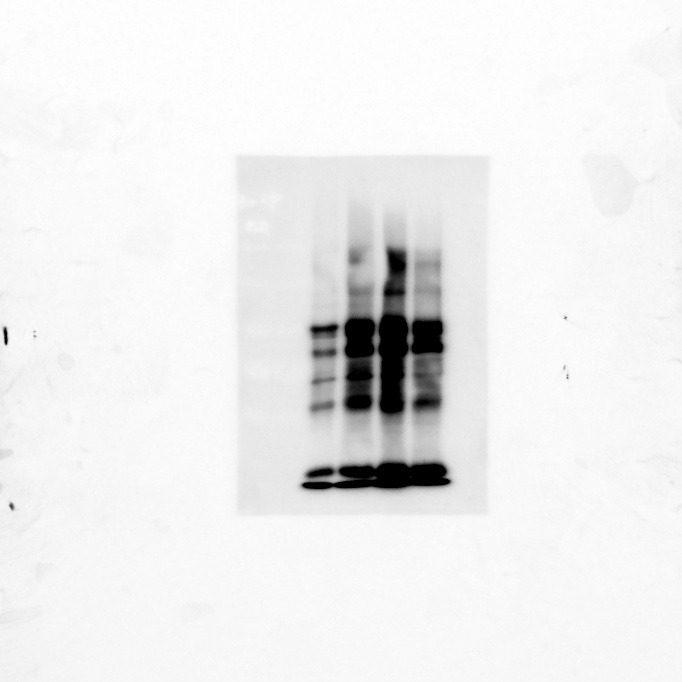

Supplement: Figure 5—source data 3. [file elife-66501-fig5-data3.zip › Figure5-source data3-Related to Figure5C/Fig5C-IP-IB-HA-Ub-K48.jpg]

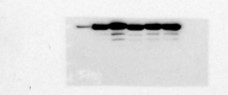

Supplement: Figure 5—source data 4. [file elife-66501-fig5-data4.zip › Figure5-source data4-Related to Figure5D/Fig5D-Input-IB-Flag.jpg]

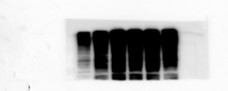

Supplement: Figure 5—source data 4. [file elife-66501-fig5-data4.zip › Figure5-source data4-Related to Figure5D/Fig5D-Input-IB-HA.jpg]

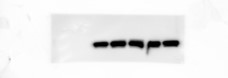

Supplement: Figure 5—source data 4. [file elife-66501-fig5-data4.zip › Figure5-source data4-Related to Figure5D/Fig5D-Input-IB-V5.jpg]

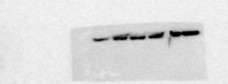

Supplement: Figure 5—source data 4. [file elife-66501-fig5-data4.zip › Figure5-source data4-Related to Figure5D/Fig5D-Input-IB-a┬-actin-MG132.jpg]

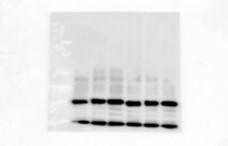

Supplement: Figure 5—source data 4. [file elife-66501-fig5-data4.zip › Figure5-source data4-Related to Figure5D/Fig5D-IP-IB-Flag.jpg]

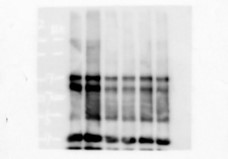

Supplement: Figure 5—source data 4. [file elife-66501-fig5-data4.zip › Figure5-source data4-Related to Figure5D/Fig5D-IP-IB-HA.jpg]

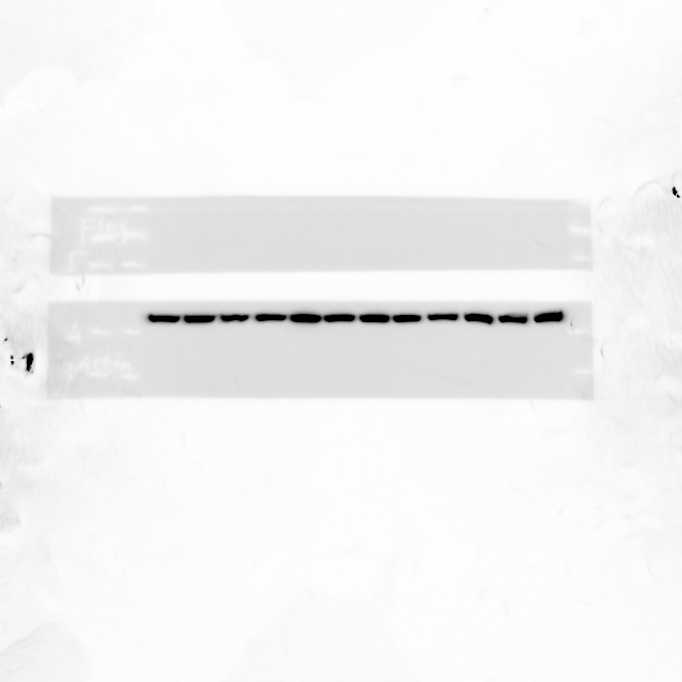

Supplement: Figure 5—source data 5. [file elife-66501-fig5-data5.zip › Figure5-source data5-Related to Figure5E/Fig 5E Actin.jpg]

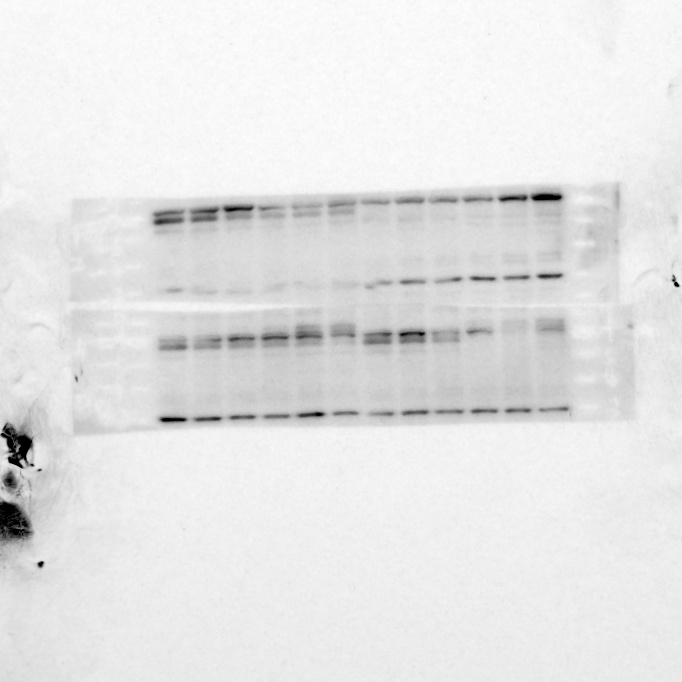

Supplement: Figure 5—source data 5. [file elife-66501-fig5-data5.zip › Figure5-source data5-Related to Figure5E/Fig 5E-Flag.jpg]

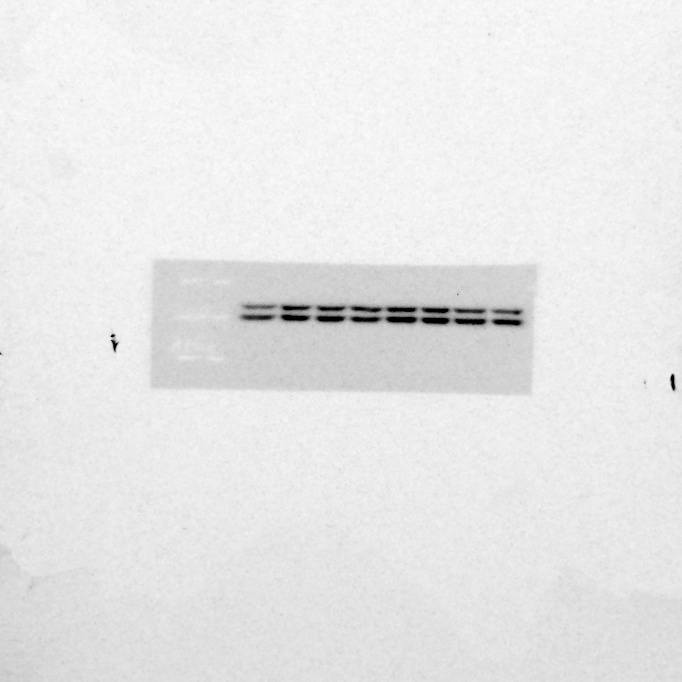

Supplement: Figure 6—source data 1. [file elife-66501-fig6-data1.zip › Figure6-source data1-Related to Figure6A/Fig6A- ERK.jpg]

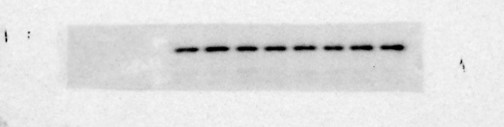

Supplement: Figure 6—source data 1. [file elife-66501-fig6-data1.zip › Figure6-source data1-Related to Figure6A/Fig6A-elf2a.jpg]

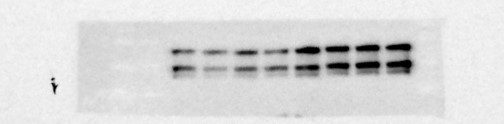

Supplement: Figure 6—source data 1. [file elife-66501-fig6-data1.zip › Figure6-source data1-Related to Figure6A/Fig6A-NF90.jpg]
