# Supplementary figures and images for: Ubiquitination and degradation of NF90 by Tim-3 inhibits antiviral innate immunity (part 2 of 2)
Source: eLife. 2021 Jun 10;10:e66501. doi: 10.7554/eLife.66501 (PMC8225388; doi:10.7554/eLife.66501)

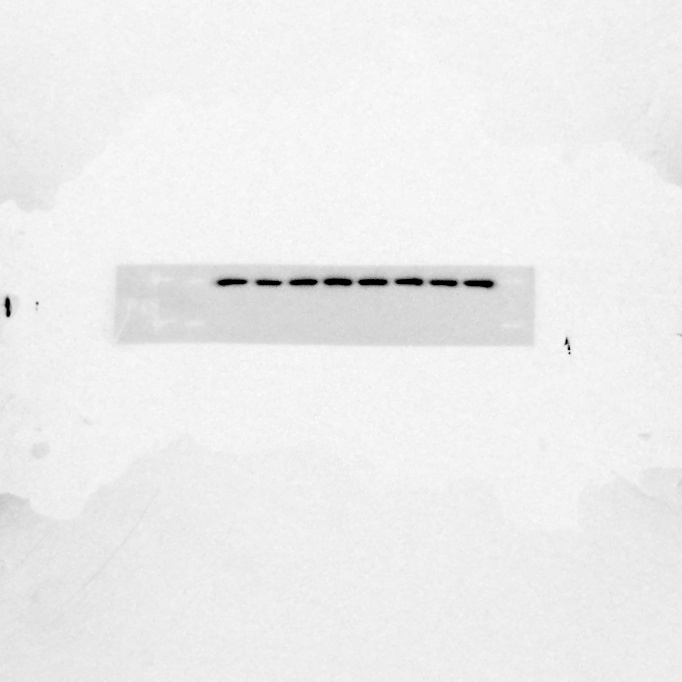

Supplement: Figure 6—source data 1. [file elife-66501-fig6-data1.zip › Figure6-source data1-Related to Figure6A/Fig6A-P38.jpg]

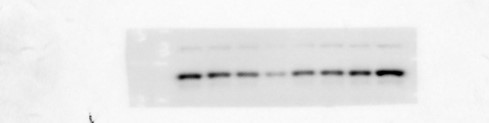

Supplement: Figure 6—source data 1. [file elife-66501-fig6-data1.zip › Figure6-source data1-Related to Figure6A/Fig6A-p-elf2a.jpg]

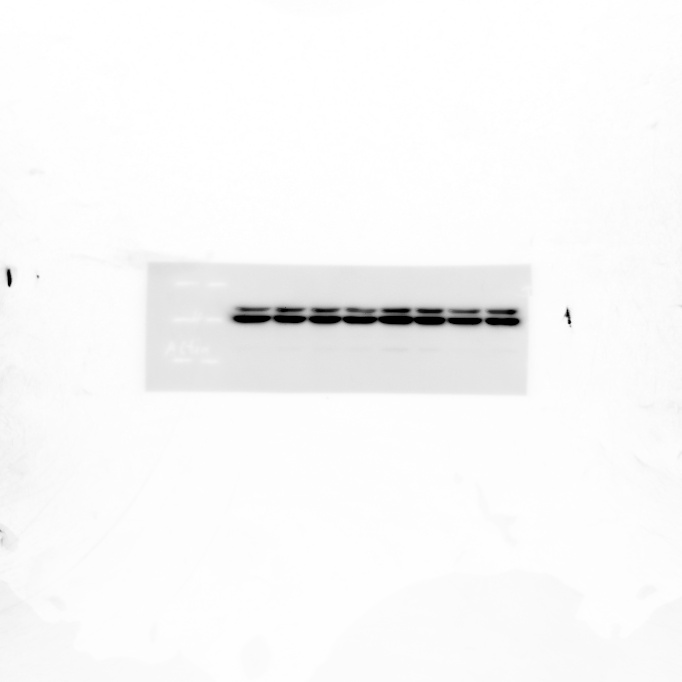

Supplement: Figure 6—source data 1. [file elife-66501-fig6-data1.zip › Figure6-source data1-Related to Figure6A/Fig6A-p-ERK.jpg]

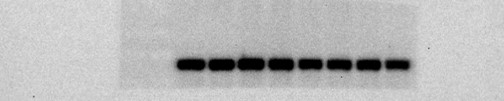

Supplement: Figure 6—source data 1. [file elife-66501-fig6-data1.zip › Figure6-source data1-Related to Figure6A/Fig6A-PKR.jpg]

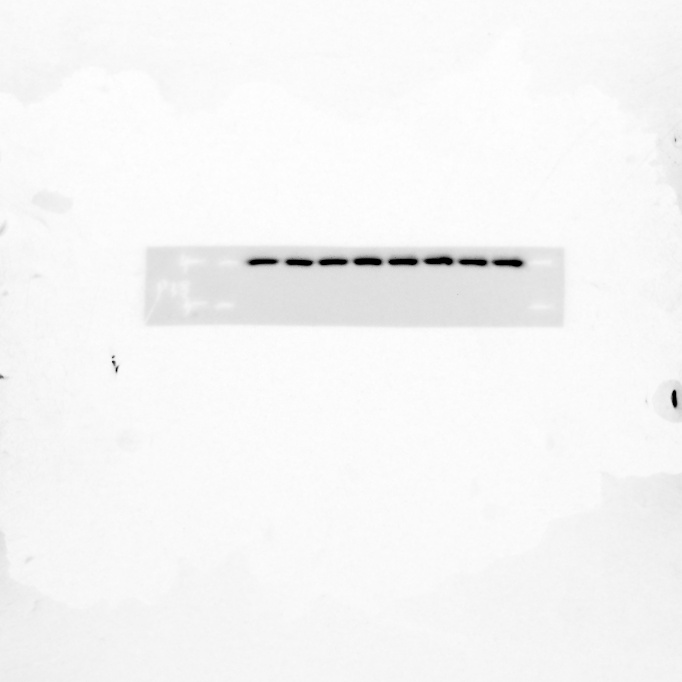

Supplement: Figure 6—source data 1. [file elife-66501-fig6-data1.zip › Figure6-source data1-Related to Figure6A/Fig6A-p-P38.jpg]

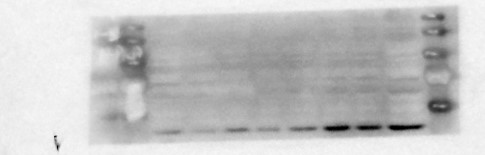

Supplement: Figure 6—source data 1. [file elife-66501-fig6-data1.zip › Figure6-source data1-Related to Figure6A/Fig6A-P-PKR.jpg]

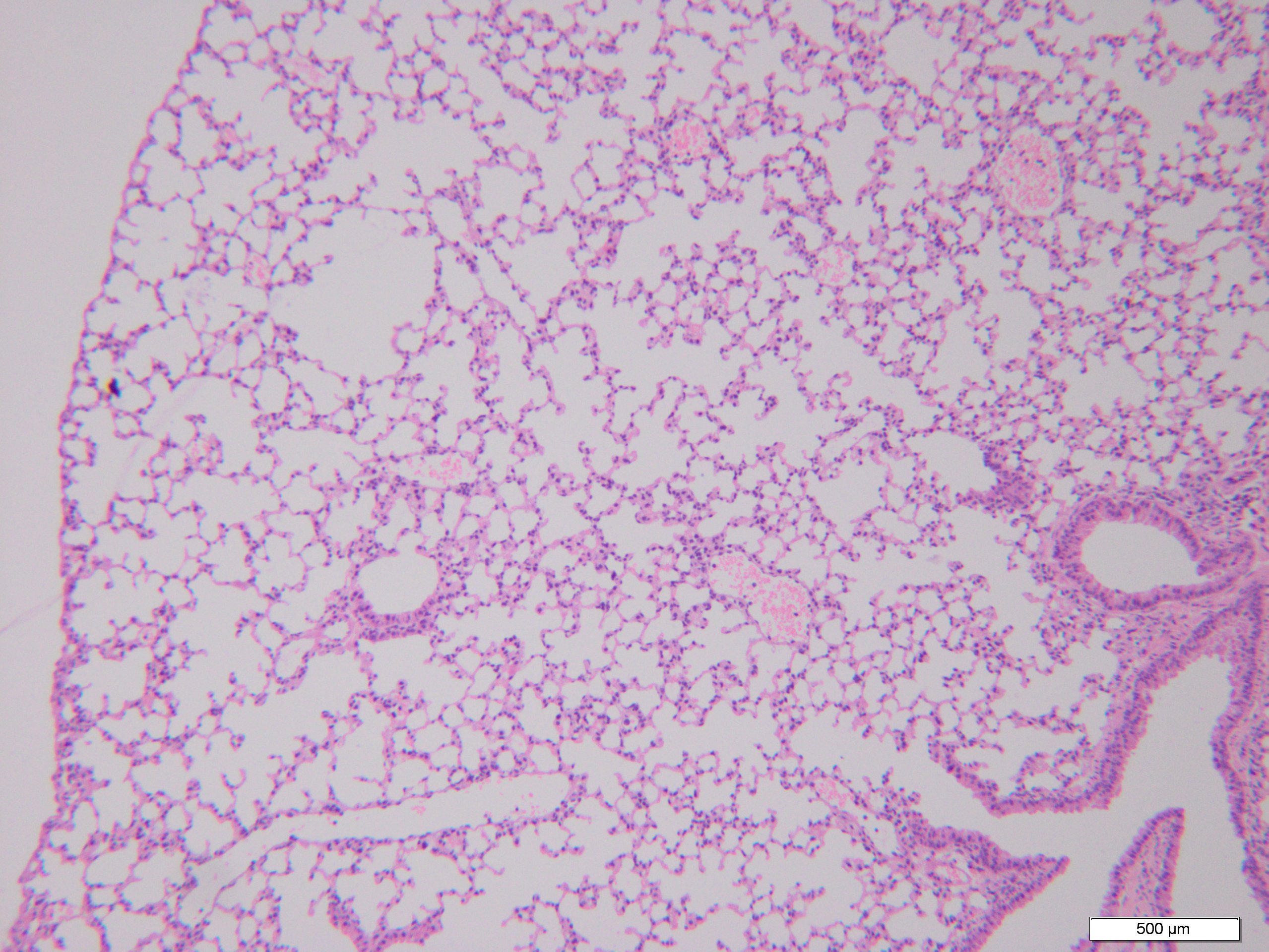

Supplement: Figure 7—source data 11. [file elife-66501-fig7-data11.zip › Figure7-source data11-Related to Figure7K/Fig.7K/Image_27815 Tim-3++ PBS 10.jpg]

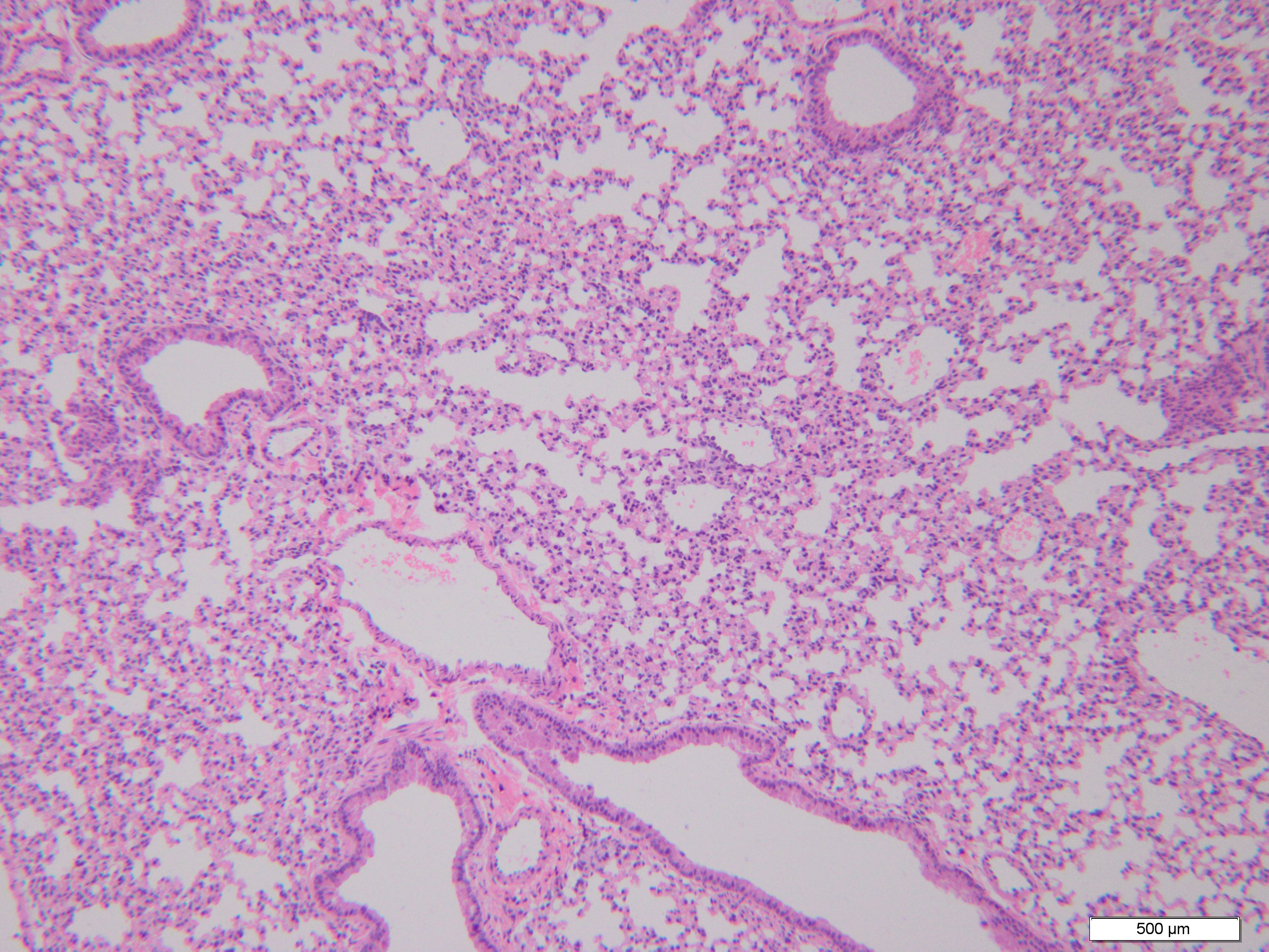

Supplement: Figure 7—source data 11. [file elife-66501-fig7-data11.zip › Figure7-source data11-Related to Figure7K/Fig.7K/Image_27823 Tim-3++ VSV 10.jpg]

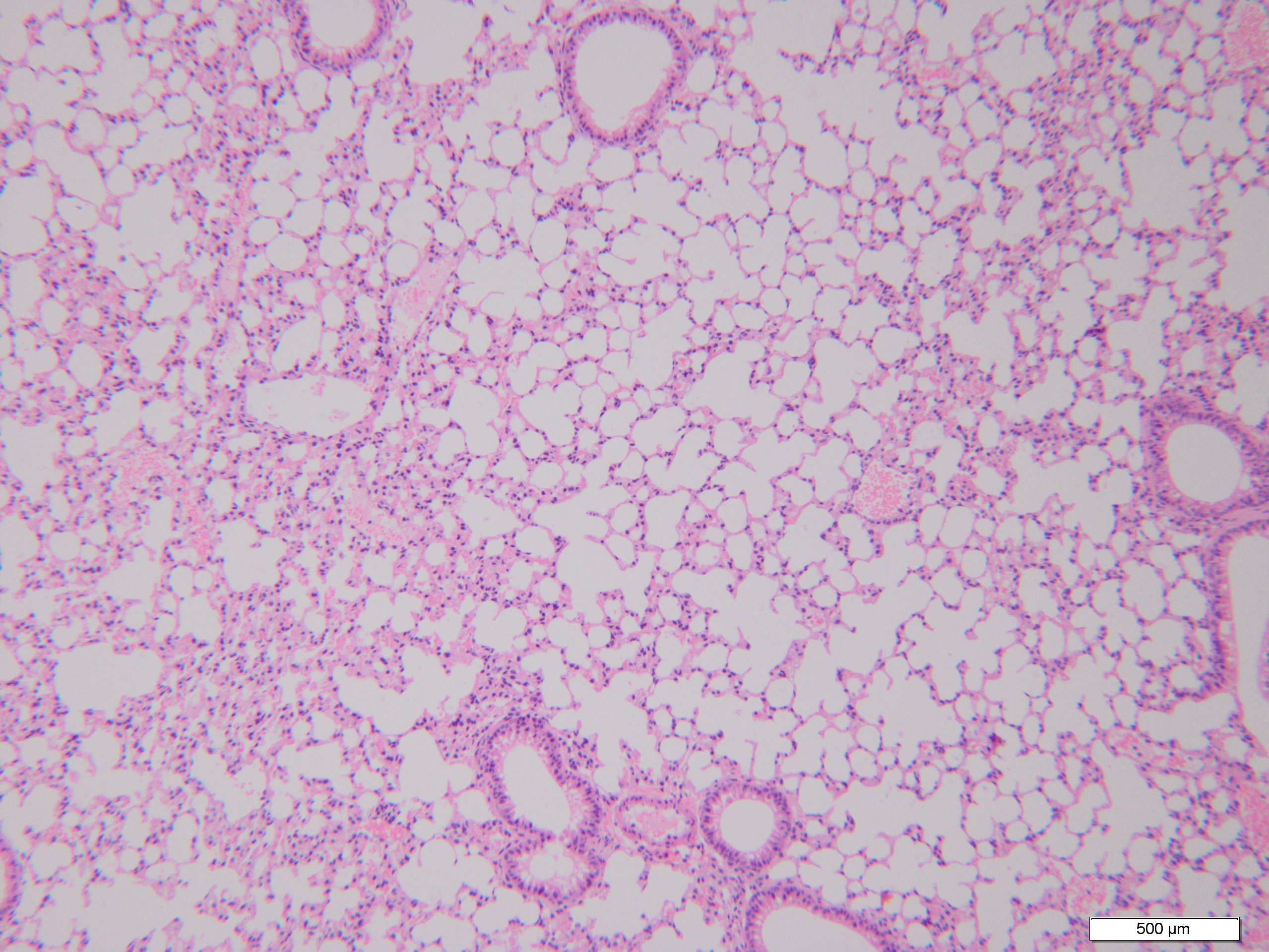

Supplement: Figure 7—source data 11. [file elife-66501-fig7-data11.zip › Figure7-source data11-Related to Figure7K/Fig.7K/Image_27831 Tim-3-- PBS 10.jpg]

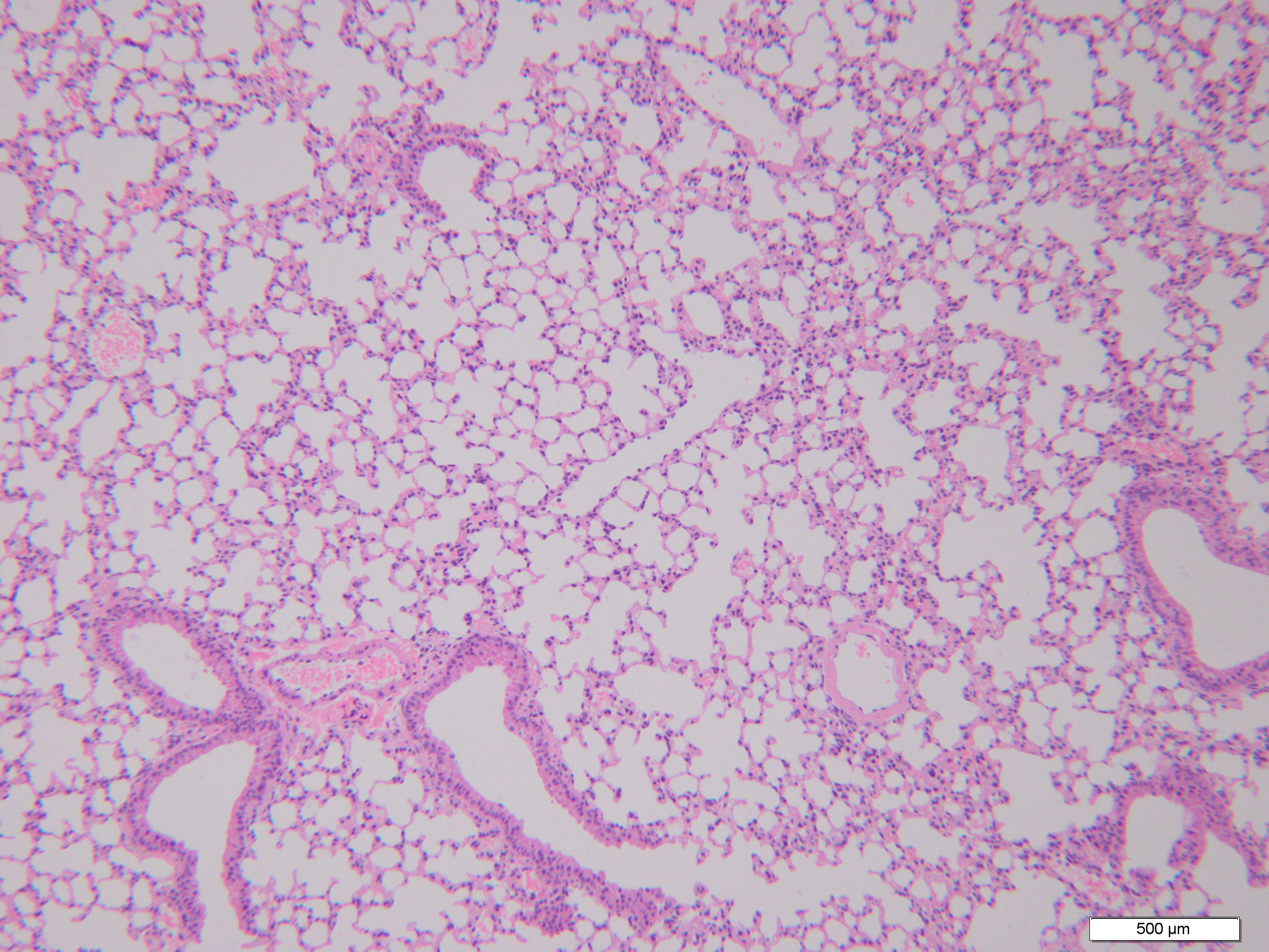

Supplement: Figure 7—source data 11. [file elife-66501-fig7-data11.zip › Figure7-source data11-Related to Figure7K/Fig.7K/Image_27839 Tim-3-- VSV 10.jpg]
