# Supplementary material for: Ubiquitination and degradation of NF90 by Tim-3 inhibits antiviral innate immunity
Source: eLife. 2021 Jun 10;10:e66501. doi: 10.7554/eLife.66501 (PMC8225388; doi:10.7554/eLife.66501)
Supplement: Supplementary file 1. [file elife-66501-supp1.docx]

**Supplementary Table**

**Table 1. Sequences of the primers used for PCR**

|  | Sense primer | Antisense primer |
| --- | --- | --- |
| 18S | 5’- TTGACGGAAGGGCACCACCAG -3’ | 5’- GCACCACCACCACGGAATCG -3’ |
| GAPDH | 5’- AACTTTGGCATTGTGGAAGG -3’ | 5’- ACACATTGGGGGTAGGAACA -3’ |
| TIA-1 | 5’- CAGTTCCCATGAAAGTGCAGC -3’ | 5’- CCACATGCCCTTCAATGGTAGTA -3’ |
| G3BP1 | 5’- AAATGGAAGGTACGCTTCACAG -3’ | 5’- CCACTGCCAAAACTGCCAAC -3’ |
| TRIM47 | 5’- CAGAAACTCGGCTCAGAAGCA -3’ | 5’- ACGATGTAGGCAAACTTGAGGA -3’ |
| VSV | 5’- ACGGCGTACTTCCAGATGG -3’ | 5’- CTCGGTTCAAGATCCAGGT -3’ |
| Tim-3 | 5’- CAGGTCTTACCCTCAACTGTG -3’ | 5’- GGCAGATAGGCATTTTTACCA -3’ |
